# Pericardial synovial sarcoma in a dyspnoeic female with tuberculous pericarditis: A case report

**DOI:** 10.3892/ol.2013.1279

**Published:** 2013-04-02

**Authors:** XIA WU, RAN CHEN, BOWEN ZHAO

**Affiliations:** 1 Departments of Radiology, Sir Run Run Shaw Hospital, Zhejiang University College of Medicine and Sir Run Run Shaw Institute of Clinical Medicine, Hangzhou, Zhejiang 310016, P.R. China; 2 Diagnostic Ultrasound and Echocardiography, Sir Run Run Shaw Hospital, Zhejiang University College of Medicine and Sir Run Run Shaw Institute of Clinical Medicine, Hangzhou, Zhejiang 310016, P.R. China

**Keywords:** heart, pericardium, synovial sarcoma

## Abstract

Synovial sarcomas of the pericardium are extremely rare and associated with poor survival rate. The current case report describes a 45-year-old female who presented with dyspnea upon exertion, a paroxysmal cough, night sweats and recurrent pericardial effusion. The patient was diagnosed with tuberculous pericarditis and received antituberculous drug therapy. Echocardiography and magnetic resonance imaging (MRI) revealed a pericardial mass lying predominantly over the right atrium. The patient was treated by surgical excision and a subsequent histological analysis confirmed the diagnosis of a pericardial synovial sarcoma. Under high power examination, a characteristic biphasic appearance consisting of hypercellular spindled cell sheets was observed. Immunohistochemistry demonstrated positive staining for epithelial membrane antigen (EMA), vimentin and Bcl 2. The patient was then referred for adjuvant chemotherapy with a combination of adriamycin and ifosfamide. The patient has since remained clinically free of disease for 32 months.

## Introduction

Primary cardiac sarcomas are extremely rare, with angiosarcoma, fibrosarcoma, rhabdomyosarcoma and malignant fibrous histiocytoma occurring in order of decreasing frequency (1). Synovial sarcomas arise from mesenchymal tissue, which differentiates sufficiently to have the histological appearance of synovium. The majority (80–95%) of tumors are reported in the extremities of young adults, with two-thirds being located in the lower limbs. Other sites of origin include the head and neck, paravertebral region, chest and abdominal wall (2). At present, few cases of synovial sarcoma that occur in pericardium have been published in the literature (1,3–8). Pericardial synovial sarcoma is associated with an extremely poor survival rate with a disease-free survival of 12 months after surgery (1), and an overall survival of 7 months (9). Due to its rarity, there are no available treatment guidelines for this disease. The present case report describes a case of pericardial synovial sarcoma that was treated with surgery and adjuvant chemotherapy. Informed consent was obtained from the patient.

## Case report

### Patient

**Figure 1:**
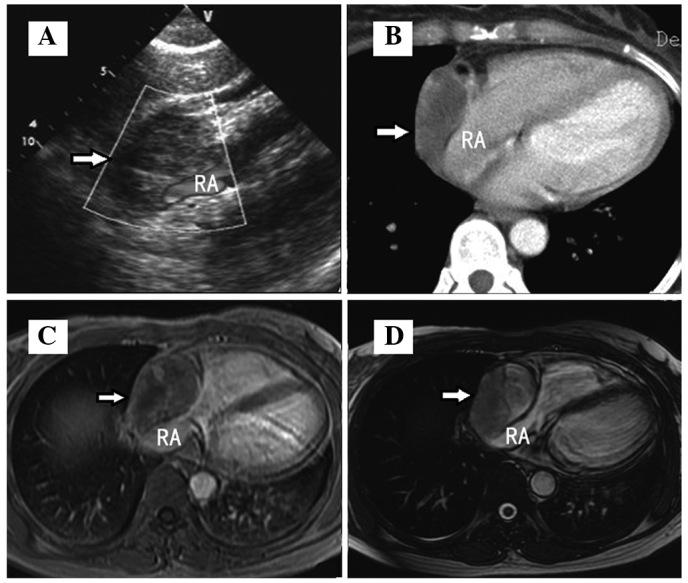
Echocardiography and radiological images of pericardial synovial sarcoma. (A) Echocardiography revealed a mass (arrow) within the pericardial space predominantly over the right atrium. (B) Contrast-enhanced computed tomography (CT) revealed a low-attenuation lesion (arrow) in the pericardium, with inhomogeneous peripheral enhancement. (C) T1-weighted post-gadolinium magnetic resonance imaging (MRI) identified mild heterogeneous enhancement (arrow). (D) 2D FIESTA sequence images revealed a 3.4×5.2-cm, high-signal, heterogeneous multilocular mass (arrow). FIESTA, fast imaging employing steady-state acquisition; RA, right atrium.

A 45-year-old female presented in January 2010 with progressive dyspnea upon exertion, a paroxysmal cough and night sweats. The patient was diagnosed with tuberculous pericarditis and received antituberculous drug therapy. A physical examination revealed quiet heart sounds. Echocardiography (iE33; Philips Electronics, Amsterdam, The Netherlands) revealed a mass measuring 3.8×5.2 cm within the pericardial space and predominantly over the right atrium, as well as a pericardial effusion. Contrast-enhanced computed tomography (CT; Siemens AG, Erlangen, Germany) demonstrated a low-attenuation lesion in the pericardium, with inhomogeneous peripheral enhancement. Magnetic resonance imaging (MRI; Signa Excite 1.5T, General Electric Company, Fairfield, CT, USA) revealed a 3.4×5.2-cm, high-signal, heterogeneous, multilocular mass on 2D fast imaging employing steady-state acquisition (FIESTA) sequence images. The lesion was present in the pericardium adjacent to the right atrium, which was significantly extruded. T1-weighted post-gadolinium imaging identified mild heterogeneous enhancement (Fig. 1). No positive mediastinal nodes were detected.

### Surgery

A thoracotomy was performed and revealed marked soft pericardial adhesions. The tumor was located between the pericardial serous layer and the right atrium, arising from the junction of the inferior vena cava and the pericardium. The tumor was excised and a partial pericardiectomy was performed with negative microscopic margins. The pericardial effusion cytology was negative for malignant cells.

### Tumor characteristics

**Figure 2:**
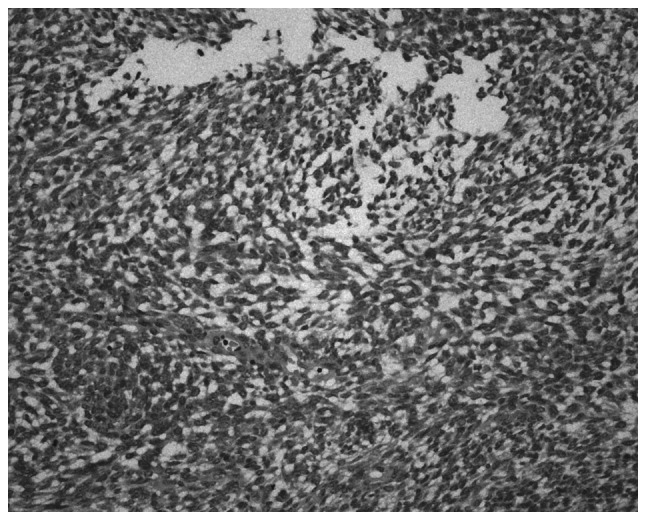
Photomicrograph revealing pleomorphic spindle-shaped cells with abundant mitotic activity (hematoxylin and eosin stain; magnification, ×20).

The excised mass measured ∼5×6 cm and had a friable texture consisting of blood clots and necrotic tissue. Microscopically, the tumor exhibited a characteristic biphasic appearance consisting of hypercellular spindled-cell sheets. Hemorrhaging, necrosis, heteromorphism and Allotypic nuclear division were also noted (Fig. 2). Immunohistochemistry demonstrated positive staining for epithelial membrane antigen (EMA), vimentin and Bcl-2, but negative staining for CD99, CD34, CD68, S100, cytokeratin, desmin, calretinin, HMBE-1, CK5/6 and smooth muscle actin, confirming a diagnosis of biphasic synovial sarcoma. One and a half months later, the patient was referred for adjuvant chemotherapy with a combination of adriamycin (20 mg/m^2^, 3 times every 3 weeks) and ifosfamide (3 g/m^2^/d on 3 subsequent days, 3-week intervals).

## Discussion

Primary sarcomas of the heart are rare (10) and include a number of histopathological variants. In total, 8–10% of cardiac sarcomas are synovial sarcomas, which are histologically classified into the following 4 subtypes depending on the relative proportion of epithelial and spindle cells: (i) Biphasic, (ii) monophasic fibrous (spindle cell), (iii) monophasic epithelial and (iv) poorly differentiated. Primary pericardial synovial sarcomas are the rarest, with only 7 reports in the literature (1,3–8), and are associated with the longest survival period of 14 years (3).

In the early stages of pericardial synovial sarcoma, symptoms are usually nonspecific or slight chest tightness is noted (4). With the progression of malignant tumors, symptoms may appear, including local invasion, which may lead to arrhythmias and tamponade, and vascular invasion, which may lead to dyspnea, chest pain or heart failure. On CT and MRI, synovial sarcoma of the pericardium typically presents as a solitary bulky mass that does not infiltrate the pericardium. CT is useful for the identification of subtle soft tissue calcifications and local bony changes. In a previous study by O’Sullivan *et al*(2), MRI was considered to represent the best modality for the detection and staging of soft tissue tumors. Synovial sarcomas are usually multilocular with internal septa and are well-defined in the majority of cases. In the study by O’Sullivan *et al* Hemorrhaging was noted in >40% of the lesions, which demonstrated high-intensity signals on T1- and T2-weighted images. No difference was noted between MRI characteristics for the monophasic and biphasic pathological subtypes.

Due to the rarity of primary pericardial synovial sarcoma, there are no standard treatment guidelines currently available (3,5). However, surgical resection is widely accepted as a primary treatment. Depending on the location of the tumor, various surgical approaches may be selected. Radiotherapy is recommended for positive resection margins to reduce local recurrence rates. However, cardiac irradiation may lead to long-term cardiac damage. In the case of a strong family history of cardiovascular problems, the total dose of radiation must be restricted to avoid long-term toxicity. Notably, no standardized chemotherapy protocol is currently followed. According to a previous study, adriamycin or a combination of adriamycin and ifosfamide represent the most effective chemotherapeutic agents (3). In the present case, the combination of adriamycin and ifofamide was used with a satisfactory curative effect.

Immunohistochemical markers, including vimentin, EMA and cytokeratin, are used to aid the pathological diagnosis of synovial sarcoma (4). However, further cytogenetic analysis may also be necessary. From all the patients with synovial sarcoma, >90% have a t(X;18) translocation mutation, which has not been found to be associated with other sarcomas. This translocation involves the SSX1 or SSX2 gene on the X chromosome (Xp11) and the SYT gene on chromosome 18 (18q11). Subtypes of these translocations have been revealed to correlate with the various histological subtypes of synovial sarcoma (11).

Although primary cardiac synovial sarcoma is associated with an extremely poor survival rate, for tumors of pericardial origin, the overall survival remains unknown. In the present case, following surgical excision and post-operative adjuvant chemotherapy, the patient has remained clinically free of disease for 32 months.
